# Temporal relationship between antibiotic use and respiratory virus activities in the Republic of Korea: a time-series analysis

**DOI:** 10.1186/s13756-018-0347-8

**Published:** 2018-04-25

**Authors:** Sukhyun Ryu, Sojung Kim, Bryan I. Kim, Eili Y. Klein, Young Kyung Yoon, Byung Chul Chun

**Affiliations:** 1 Division of Infectious Disease Control, Gyeonggi Provincial Government, Suwon, Republic of Korea; 2 Department of Epidemiology and Health Informatics, Graduate School of Public Health, Korea University, Seoul, Republic of Korea; 3 Department of Insurance Benefit, National Health Insurance Service, Seoul, Republic of Korea; 4 Center for Disease Dynamics, Economics & Policy, Washington D.C., USA; 5 Department of Emergency Medicine, Johns Hopkins University, Baltimore, USA; 6 Division of Infectious Diseases, Department of Internal Medicine, Korea University College of Medicine, Seoul, Republic of Korea; 7 Department of Preventive Medicine, Korea University College of Medicine, Seoul, Republic of Korea

**Keywords:** Antibiotic use, Influenza, Respiratory virus, Korea, Time-series analysis

## Abstract

**Background:**

Inappropriate use of antibiotics increases resistance and reduces their effectiveness. Despite evidence-based guidelines, antibiotics are still commonly used to treat infections likely caused by respiratory viruses. In this study, we examined the temporal relationships between antibiotic usage and respiratory infections in the Republic of Korea.

**Methods:**

The number of monthly antibiotic prescriptions and the incidence of acute respiratory tract infections between 2010 and 2015 at all primary care clinics were obtained from the Korean Health Insurance Review and Assessment Service. The monthly detection rates of respiratory viruses, including adenovirus, respiratory syncytial virus, influenza virus, human coronavirus, and human rhinovirus, were collected from Korea Centers for Disease Control and Prevention. Cross-correlation analysis was conducted to quantify the temporal relationship between antibiotic use and respiratory virus activities as well as respiratory infections in primary clinics.

**Results:**

The monthly use of different classes of antibiotic, including penicillins, other beta-lactam antibacterials, macrolides and quinolones, was significantly correlated with influenza virus activity. These correlations peaked at the 0-month lag with cross-correlation coefficients of 0.45 (*p* < 0.01), 0.46 (*p* < 0.01), 0.40 (*p* < 0.01), and 0.35 (< 0.01), respectively. Furthermore, a significant correlation was found between acute bronchitis and antibiotics, including penicillin (0.73, *p* < 0.01), macrolides (0.74, *p* < 0.01), and quinolones (0.45, *p* < 0.01), at the 0-month lag.

**Conclusions:**

Our findings suggest that there is a significant temporal relationship between influenza virus activity and antibiotic use in primary clinics. This relationship indicates that interventions aimed at reducing influenza cases in addition to effort to discourage the prescription of antibiotics by physicians may help to decrease unnecessary antibiotic consumption.

## Background

Overuse and inappropriate use of antibiotics drive the emergence and spread of antimicrobial resistance [1, 2]. In the Republic of Korea, the number of antibiotic prescriptions is relatively higher (31.7 defined daily dose [DDD] per 1000 inhabitants per day) than in other member countries of the Organization for Economic Co-operation and Development (mean, 23.7 DDD per 1000 inhabitants per day) [3]. In Korea, the majority of antibiotics (ca. 90%) are prescribed in primary care and mainly for acute respiratory tract infections (ARTIs; ca. 57%) [4]. ARTIs are mainly viral in origin, are generally self-limiting, and do not require antibiotics [5, 6]. Secondary bacterial pneumonia is the most important clinical complication of respiratory viral infections. However, previous studies have shown that antibiotics do not improve outcomes for patients with ARTIs [7–10].

To prevent overuse and inappropriate use of antibiotics, it is essential to identify and understand antibiotic prescribing patterns and determining factors, however, little is known about antibiotic prescribing patterns in the Republic of Korea. The purpose of this study was to describe antibiotic prescription patterns in primary care clinics over a 6-year period and to identify its temporal relationship with respiratory viruses and ARTIs.

## Methods

### Antibiotic use data

The National Health Insurance covers 98% of the total Korean population, providing near- complete coverage of all antibiotic prescriptions in the Republic of Korea. Reimbursement data from over 80,000 healthcare service providers in Korea were collected from the Korean Health Insurance Review and Assessment Service (KHIRA). The data covers 46 million patients annually, approximately 90% of the population of the Republic of Korea, and includes patients’ diagnoses (recorded using the *International Classification of Diseases, Clinical Modification, 10th Revision* [ICD-10-CM]), and prescription drugs [11, 12]. We collected monthly antibiotic prescription data from primary care clinics between January 2010 and December 2015 in accordance with the Anatomic Therapeutic Chemical Classification System (J01A: tetracyclines; J01C: beta-lactam antibacterials, penicillins; J01D: other beta-lactam antibacterials [cephalosporins, monobactams, and carbapenems]; J01F:macrolides, lincosamides, and streptogramins; J01G: aminoglycosides; J01MA: fluoroquinolones). Prescription data were converted to DDD per 1000 inhabitants per day (DID), the assumed average maintenance dose per day for a prescribed medication. Population data were obtained from census data provided by Korean Statistical Information Service.

### Respiratory virus surveillance data

The number of acute respiratory virus diagnoses was collected from the Korea Influenza and Respiratory Virus Surveillance System (KINRESS) from the Korea Centers for Disease Control and Prevention. KINRESS collects nasopharyngeal specimens from patients with acute respiratory symptoms, including cough, rhinorrhea, and sore throat, from sentinel primary care clinics. This weekly laboratory-based surveillance system has been in operation since 2009 to measure respiratory virus activity at the community level, including adenovirus (ADV), influenza virus (IFV; A, B), human coronavirus (hCoV; 229E, OC43, NL63), human rhinovirus (hRV), and respiratory syncytial virus (RSV; A, B). Laboratory confirmation of respiratory pathogens was performed using multiplex polymerase chain reaction (PCR) or real-time reverse transcription PCR [13, 14].

### Incidence of acute respiratory tract infections

We obtained the monthly number of ARTI diagnoses between 2010 and 2015 from the KHIRA database using ICD-10-CM codes. All patients diagnosed with an ARTI, regardless of age or gender, were included. ARTIs were defined as acute bronchitis and acute upper respiratory tract infection. In addition, we included acute tonsillitis and pneumonia as comparators as they are more likely to require antibiotics than other ARTIs [15]. Incidence was calculated by dividing the number of ARTI diagnoses by the population of the Republic of Korea during the study period.

### Statistical analysis

We used regression analysis to describe the trends of antibiotic use, respiratory virus activity, and the incidence of ARTIs, including acute bronchitis, overall.

To identify the temporal relationship between antibiotic prescriptions and respiratory virus activity and the incidence of ARTIs, we performed a cross-correlation function test. This cross-correlation test is widely used in identifying the time lags of onetime series (respiratory virus) with the possible predictors of another time series (antibiotic use) [16, 17].

The Box-Jenkins method was applied to fit time-series data to seasonal autoregressive moving average models [18, 19]. Stationary time series was evaluated using the augmented Dickey-Fuller test to determine whether differencing is required to rule out spurious correlations. The Akaike information criterion test, the portmanteau test, and a normality check of the residuals were conducted to identify the best model fit. Cross-correlation analysis using the residuals from each time-series model was used to evaluate the temporal relationship between the antibiotic prescription rate and respiratory virus detection as well as the incidence of ARTIs.

The statistical package R, version 3.2.4 (R Foundation for Statistical Computing, Vienna, Austria) was used for all statistical analysis. All *p*-values were 2-sided and considered significant at *p* < 0.05.

## Results

### Antibiotic use

**Fig. 1:**
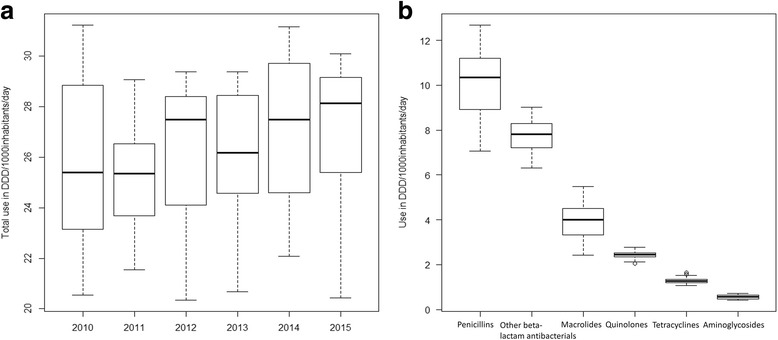
**a** Trends of total amounts of antibiotic use in primary care clinics between 2010 and 2015. **b** Average use of main antibiotic classes

The average DID of the total antibiotic prescriptions during the study period was 26.2 (range, 20.3-31.2). For primary clinics, the prescribing rate was 25.2 (range, 20.6-31. 2) DID in 2010 and 26.9 (range, 20.4-30.1) in 2015 with a tendency to increase (*p* < 0.01) (Fig. 1a).

The most commonly used classes of antibiotic were penicillin (DID range, 7.1-12.7; mean, 10.1), other beta-lactams antibacterials (DID range, 6.3-9.0; mean, 7.7), macrolides (DID range, 2.4-5.5; mean, 3.9), fluoroquinolones (DID range, 2.0-2.8; mean, 2.4), tetracyclines (DID range, 1.1-1.6; mean, 1.3), and aminoglycosides (DID range, 0.4-0.7; mean, 0.6) (Fig. 1b).

### Acute respiratory virus activities

**Fig. 2:**
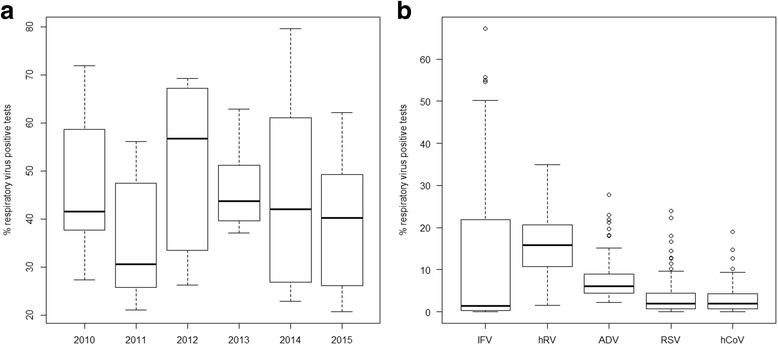
**a** Trends of overall respiratory virus activity between 2010 and 2015. **b** Average overall detection rate of respiratory viruses between 2010 and 2015

Mean annual detection rates of respiratory viruses fluctuated highly in 2011 and 2012, but was largely stable in the other years, though estimated ranges were relatively large. In 2010, 47% (range, 27-72%) of isolates had a virus, while only 39% (range, 21-62%) were detected in 2015 (Fig. 2a).

The most commonly detected respiratory viruses were hRV (range, 2-35%; median, 16%), IFV (range, 0-62%; median, 2%), ADV (range, 2-28%; median, 6%), RSV (range, 0-24; median, 2%), and hCoV (range, 0-19%; median, 2%) (Fig. 2b).

### Incidence of acute respiratory tract infections

**Fig. 3:**
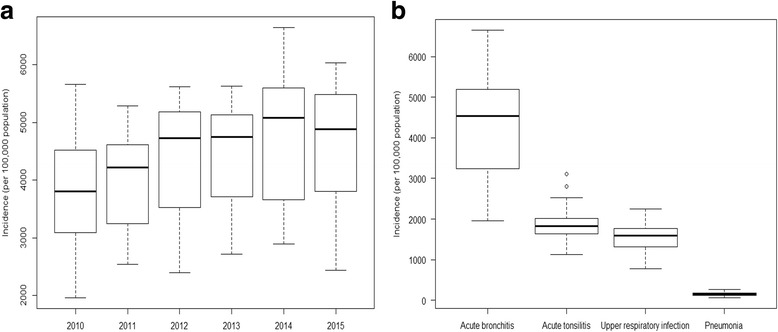
**a** Trends of overall acute respiratory tract infections between 2010 and 2015. **b** Monthly average incidences of acute respiratory infections between 2010 and 2015

The annual incidence of acute bronchitis increased significantly from 3836 (range, 1964-5665; mean, 3836) per 100,000 individuals in 2010 to 4612 (range, 2440-6034; mean, 4612) per 100,000 individuals in 2015 (*p* < 0.01) (Fig. 3a). The average incidences of acute bronchitis, acute tonsillitis, acute upper respiratory tract infections, and pneumonia were 4334, 1864, 1526, and 153 per 100,000 individuals, respectively (Fig. 3 (b)).

### Correlation analysis of antibiotic use with respiratory virus detection and incidence of respiratory infections

**Fig. 4:**
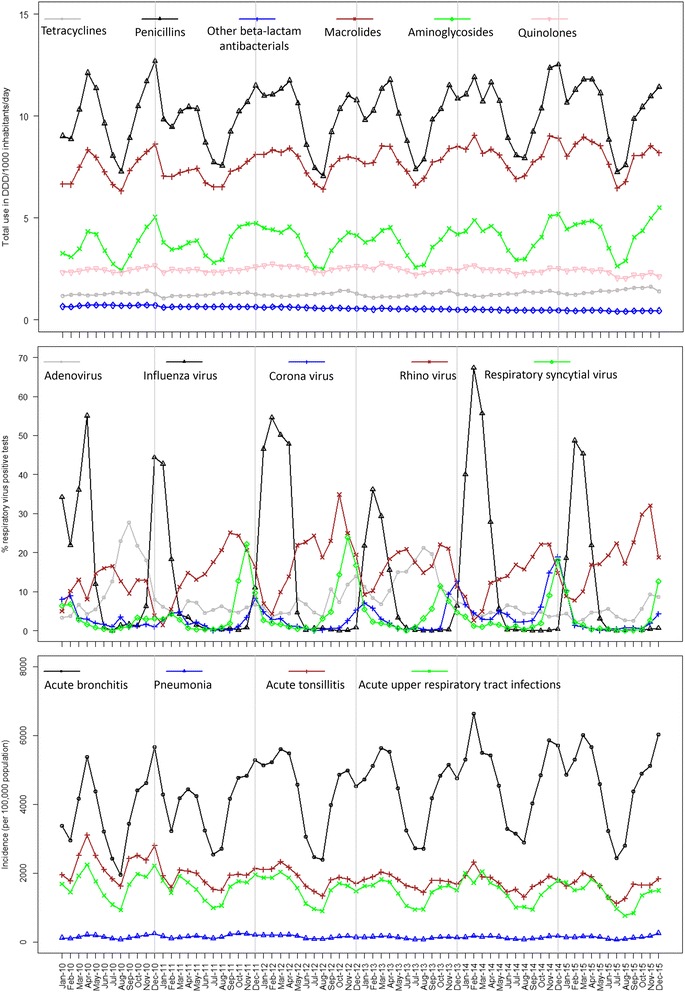
Descriptive trends of antibiotic use, respiratory virus activities, and the incidence of acute respiratory disease

Monthly time series of antibiotic use, respiratory virus detection, and incidence of ARTIs are presented in Fig. 4. Seasonal antibiotic use clearly followed a similar oscillatory pattern to influenza virus detection. Antibiotic use also had a similar seasonal pattern as the incidences of acute bronchitis, acute upper respiratory tract infections, and acute tonsillitis.

The total monthly rate of antibiotic prescriptions was highly cross-correlated with the monthly detection rate of influenza virus (cross-correlation coefficient 0.47, *p* < 0.01). In bivariate analyses, antibiotic use rates for the 4 most commonly used antibiotics (penicillins, other beta-lactam antibacterials, macrolides, and fluoroquinolones) were significantly cross-correlated with influenza virus detection at the 0-month lag with cross-correlation coefficients of 0.45 (*p* < 0.01), 0.46 (*p* < 0.01), 0.40 (*p* < 0.01), and 0.35 (< 0.01), respectively (Table 1). However, no cross-correlation was found between antibiotic classes with lower-use rates (< 2 DID) and the influenza virus detection rate. There was significant cross-correlation between hRV and tetracycline with a 2-month lag (cross-correlation coefficient 0.24, *p* = 0.04).

**Table 1:** Cross-correlation coefficients between antibiotic use and respiratory viruses (2010-2015)

Antibiotics	RSV	IFV	hCoV	hRV	ADV
Tetracyclines	−0.14 *p* = 0.24 0-month lag	0.05 *p* = 0.67 7-month lag	−0.09 *p* = 0.43 − 3-month lag	0.24 *p* = 0.04 2-month lag	− 0.07 *p* = 0.55 0-month lag
Penicillins	− 0.01 *p* = 0.98 10-month lag	0.45 *p* < 0.01 0-month lag	0.06 *p* = 0.64 2-month lag	−0.13 *p* = 0.29 2-month lag	− 0.09 *p* = 0.44 6-month lag
Other beta-lactam antibacterials	−0.02 *p* = 0.90 1-month lag	0.46 *p* < 0.01 0-month lag	0.02 *p* = 0.85 1-month lag	−0.14 *p* = 0.24 2-month lag	−0.11 *p* = 0.34 0-month lag
Macrolides	0.12 *p* = 0.31 10-month lag	0.40 *p* < 0.01 0-month lag	−0.06 *p* = 0.60 1-month lag	−0.08 *p* = 0.50 8-month lag	− 0.16 *p* = 0.18 9-month lag
Aminoglycosides	−0.03 *p* = 0.79 10-month lag	0.15 *p* = 0.20 0-month lag	0.11 *p* = 0.36 1-month lag	0.10 *p* = 0.39 0-month lag	−0.11 *p* = 0.37 5-month lag
Fluoroquinolones	−0.02 *p* = 0.84 3-month lag	0.35 *p* < 0.01 0-month lag	−0.03 *p* = 0.83 2-month lag	−0.04 *p* = 0.76 2-month lag	− 0.16 *p* = 0.19 6-month lag

*Abbreviations*: *ADV* adenovirus, *hCoV* human coronavirus, *hRV* human rhinovirus, *IFV* Influenza virus, *RSV* respiratory syncytial virus

For ARTIs, the correlation coefficiencts of antibiotic use and the incidence of acute bronchitis were 0.73 (*p* < 0.01) for penicillins, 0.69 (*p* < 0.01) for other beta-lactam antibacterials, 0.74 (*p* < 0.01) for macrolides and 0.45 (*p* < 0.01) for fluoroquinolones (Table 2). Acute upper respiratory infection was significantly correlated with penicillins (0.33, *p* < 0.01), other beta-lactam antibacterials (0.32, *p* < 0.01), macrolides (0.24, *p* = 0.04), and fluoroquinolones (0.31, *p* < 0.01) without a lag. Again, no cross-correlation was found between classes of antibiotics with lower-use rates (< 2 DID) and ARTIs.

**Table 2:** Cross-correlation coefficients between antibiotic use and acute respiratory tract infections (2010-2015)

Antibiotics	Acute bronchitis	Acute tonsillitis	Acute upper respiratory infection	Pneumonia
Tetracyclines	0.44 *p* = 0.66 0-month lag	0.11 *p* = 0.34 0-month lag	0.03 *p* = 0.97 −10-month lag	−0.01 *p* = 0.90 0-month lag
Penicillins	0.73 *p* < 0.01 0-month lag	0.69 *p* < 0.01 0-month lag	0.33 *p* < 0.01 0-month lag	0.36 *p* < 0.01 0-month lag
Other beta-lactam antibacterials	0.69 *p* < 0.01 0-month lag	0.68 *p* < 0.01 0-month lag	0.32 *p* < 0.01 0-month lag	0.25 *p* = 0.03 0-month lag
Macrolides	0.74 *p* < 0.01 0-month lag	0.59 *p* < 0.01 0-month lag	0.24 *p* = 0.04 0-month lag	0.53 *p* < 0.01 0-month lag
Aminoglycosides	0.14 *p* = 0.28 0-month lag	0.20 *p* = 0.09 −3-month lag	−0.04 *p* = 0.75 0-month lag	0.38 *p* < 0.01 0-month lag
Fluoroquinolones	0.45 *p* < 0.01 0-month lag	0.35 *p* < 0.01 0-month lag	0.31 *p* < 0.01 0-month lag	0.23 *p* = 0.05 0-month lag

For comparators that were more likely to require antibiotics than ARTIs, pneumonia was significantly correlated with penicillins (0.36, *p* < 0.01), macrolides (0.53, *p* < 0.01), aminoglycosides (0.38, *p* < 0.01), and other beta-lactam antibacterials (0.25, *p* < 0.03) without a lag. Furthermore, acute tonsillitis was significantly correlated with penicillin (0.69. *p* < 0.01), other beta-lactam antibacterials (0.68, *p* < 0.01), macrolides (0.59, *p* < 0.01), and fluoroquinolones (0.35, *p* < 0.01) without a lag.

## Discussion

Our study is the first to identify the temporal relationship between the number of monthly antibiotic prescriptions and the detection rates of respiratory viruses and ARTIs in the Republic of Korea. Our results suggest that seasonal variation in the numbers of commonly prescribed antibiotics (penicillins, other beta-lactam antibacterials, macrolides, and fluoroquinolones) was significantly associated with the change in the activity of influenza in the community. Seasonal variation of antibiotic prescriptions has been documented in the United States [17, 20], Canada [21], and Europe [22]. Furthermore, it has also been shown that the incidence of influenza is highly correlated with the seasonal pattern of antibiotic prescriptions and that changes in testing can affect prescription rates of antibiotics [23–26]. These previous findings support our results that the seasonality of antibiotic use is significantly associated with influenza virus activity in the country.

Aside from the correlation between hRV and tetracycline, other viruses were not significantly correlated with antibiotic use. This may be due to the low numbers of antibiotic use against hRV.

Regarding the cross-correlation between influenza virus activity and the incidence of pneumonia, no significant temporal relationship was found within a 1-month lag (β = 0.23, *p* = 0.05). This is likely because of the low incidence of pneumonia (153 cases per 100,000 persons). This is not surprising as pneumonia is an uncommon diagnosis in the outpatient setting compared to acute bronchitis (4334 cases per 100,000 persons). Pneumonia is a quite severe infection and often requires hospitalization for confirmation. Thus, many acute bronchitis prescriptions might reflect uncertainty on the clinician part as to whether the patient may have pneumonia, and are prescribing out of an abundance of caution despite the potential downside consequences of unnecessary antibiotic use.

Regarding the relationship between influenza virus activity and the incidence of acute tonsillitis, a significant cross-correlation was found (β = 0.29, *p* = 0.01) at the 0-month lag. This result is consistent with previous literature documenting the most common cause of tonsillitis is viral infection including influenza virus [27]. Conservative management is the main treatment option for patients with tonsillitis except in the case of streptococcal infections (detection rate in Korea: 8.3%) [27]. Our results demonstrated that antibiotic use was significantly correlated with acute tonsillitis with a larger magnitude than influenza virus activity. This correlation likely results from the physician’s anxiety over the potential risk of developing secondary bacterial infections. Antibiotics prescribed for respiratory viruses are positively associated with poor quality prescribing [24, 28]. Patient satisfaction has been shown to be a major driver as well. Even patients who received a delayed antibiotic prescription were less likely to be satisfied with treatment than those who immediately received a prescription, even if the treatment outcomes were not different [29, 30]. This underlying situation may have contributed to the high rates of antibiotic use for diagnoses that generally do not require antibiotics.

Our results further suggest that antibiotic use could be lowered by reducing influenza transmission or through education campaigns aimed at the public and physicians to discourage inappropriate prescribing of antibiotics, particularly during influenza season [31]. Moreover, increasing vaccine coverage, which covers only approximately 43% of the Korean population, may reduce unnecessary antibiotic use [32]. Improved point-of-care tests for detecting influenza virus may also be likely to reduce antibiotic use [25, 26].

Our findings are subject to several limitations. First, our study is ecological and utilizes population-level data and thus may not represent associations at the individual level. Nonetheless, the significant relationship between the overuse of antibiotics and influenza virus circulation was also observed in a previous cohort study [33]. Second, since enterovirus (mean detection rate: 3.1%) has not been assessed in the KINRESS since 2011 and the mean detection rates of other respiratory viruses, such as human metapneumovirus (hMPV), human bocavirus (hBoV), and human parainfluenza virus (hPIV) were relatively low (hMPV: 1.28%, hBoV: 1.6%, hPIV: 4.0%), these viruses were not considered in this study. Third, we used primary care clinic-based sentinel surveillance data for respiratory virus detection. These data could underestimate the strength of virus activity; however, the pattern of influenza virus activity was similar to the pattern of influenza-like-illness in the country. Fourth, the number of samples collected was not consistent year on year due to the variation of respiratory virus activity (yearly mean number of samples collected is 12,938). Fifth, ARTIs may include other infectious diseases requiring antibiotic treatment, such as bacterial pneumonia.

## Conclusions

Our study identified a strong temporal association between antibiotic use, and influenza virus activity, and the incidence of ARTIs. We detected a significant correlation between antibiotic use of common antibiotics (penicillins, other beta-lactam antibacterials, macrolides, and fluoroquinolones) and influenza virus activity as well as the incidence of acute bronchitis and acute upper respiratory tract infections. Our results indicate that interventions aimed at reducing influenza infections and discouraging the use of antibiotics by physicians and the public may help to decrease antibiotic consumption. Additional studies, including precise evaluations of the Korean Influenza National Immunization Program, on antibiotic prescription patterns, may identify additional opportunities to reduce antibiotic prescriptions.

## Abbreviations

ADV: Adenovirus
ARTIs: Acute respiratory tract infections
DDD: Defined daily dose
DID: Defined daily dose per 1000 inhabitants per day
hBoV: Human bocavirus
hCoV: Human coronavirus
hMPV: Human metapneumovirus
hPIV: Human parainfluenza virus
hRV: Human rhinovirus
ICD-10-CM: International Classification of Diseases, Clinical Modification, Tenth Revision
IFV: Influenza virus
KHIRA: Korean Health Insurance Review and Assessment Service
KINRESS: Korea Influenza and Respiratory Virus Surveillance System
RSV: Respiratory syncytial virus
